# Application of three-dimensional-printed porous tantalum cones in total knee arthroplasty revision to reconstruct bone defects

**DOI:** 10.3389/fbioe.2022.925339

**Published:** 2022-09-05

**Authors:** Yunong Ao, Lin Guo, Hao Chen, Rui He, Pengfei Yang, Dejie Fu, Lingchuan Gu, Yang Peng, Ran Xiong, Liu Yang, Fuyou Wang

**Affiliations:** Center for Joint Surgery, Southwest Hospital, Third Military Medical University (Army Medical University), Chongqing, China

**Keywords:** 3D, printed, porous tantalum, bone defect, knee arthroplasty revision, reconstruction

## Abstract

**Purpose:** Three-dimensional (3D) printing technology has emerged as a new treatment method due to its precision and personalization. This study aims to explore the application of a 3D-printed personalized porous tantalum cone for reconstructing the bone defect in total knee arthroplasty (TKA) revision.

**Methods**: Between November 2017 and October 2020, six patients underwent bone reconstruction using 3D-printed porous tantalum cones in TKA revision. The knee function was assessed using the Hospital for Special Surgery (HSS) score pre- and postoperatively. The pain was measured by the visual analog scale (VAS) pre- and postoperatively. The quality of life was measured using the 36-Item Short Form Health Survey (SF-36) to pre- and postoperatively evaluate the relief of pain. Operation time, intraoperative blood loss, postoperative drainage volume, and complications were also recorded. At the last follow-up, all patients received X-ray and computed tomography (CT) to confirm the effect of bone reconstruction.

**Results:** After an average follow-up duration of 26.3 months, no patients developed any operation-related complications. The average intraoperative blood loss and postoperative drainage volumes were 250.1 ± 76.4 ml and 506.7 ± 300.8 ml, respectively. At the last follow-up, the HSS score was significantly higher than that before operation, indicating that the knee function was significantly improved (*p* < 0.001). During the follow-up, the mean VAS score decreased and the mean SF-36 score increased, both of which were significantly improved compared with preoperative conditions (*p* < 0.001). Radiological examination at the final follow-up showed that cones implanted into the joint were stable and bone defects were effectively reconstructed.

**Conclusion:** This study demonstrated that 3D-printed porous tantalum cones could effectively reconstruct bone defects and offer anatomical support in TKA revision. Further studies are still needed to confirm the long-term effect of 3D-printed tantalum cones for reconstructing bone defects.

## Introduction

Total knee arthroplasty (TKA) is an effective method for the treatment of severe osteoarthritis, rheumatoid arthritis, and various knee deformities, which can effectively relieve joint pain and reconstruct the function of the knee. Since its first clinical application in the 1960s, with the rapid update of the prosthesis design concept and the development of science and technology such as material science and bionics, the process of TKA has also been continuously improved (Fei et al., 2022; Kirschbaum et al., 2022). So far, TKA has solved various symptoms of patients with end-stage knee osteoarthritis. However, with the wide application of TKA in the clinic, the number of TKA revision is also significantly increasing year by year due to periprosthetic joint infection, aseptic loosening, polyethylene wear, osteolysis, instability, stiffness, and periprosthetic fracture, which poses great challenges to joint surgeons. According to the reports of the American Association of Orthopedic Surgeons, by 2030, the number of patients who have undergone TKA revision in the United States will reach 268,000 (Kurtz S. et al., 2007). In knee revision cases, many patients suffer from loosening of the implanted prosthesis due to metaphyseal bone defects caused by various reasons, which seriously affects the joint activity and quality of life of patients (Kurtz S. M. et al., 2007).

At present, the Anderson Orthopedic Research Institute (AORI) system is mainly used to classify metaphyseal bone defects in TKA revision. In detail, Type I has minor local cancellous bone defects which do not affect joint stability. Type II defects are mainly divided into two categories: type IIA only involves one tibial plateau or femoral condyle, while type IIB involves the entire tibial plateau or two femoral condyles (Rossi et al., 2022). Type III defects have severe bone loss and involve a wide range and are often accompanied by collateral ligament injury. The traditional treatment methods for bone defects during TKA revision mainly include bone cement filling, structural allografts, and metal cones (Ritter and Harty, 2004; Tsukada et al., 2013; Lei et al., 2019).

However, the specific bone defect in each patient is extremely complex, and the shape of the bone defect varies widely. The aforementioned treatments cannot effectively reconstruct bone defects, and it is, especially difficult to obtain an ideal therapeutic effect for giant bone defect (Daines and Dennis, 2012; Bloch et al., 2020). Three-dimensional (3D) printing provides a new treatment strategy for bone defects in TKA revision, designing personalized prostheses based on radiographic data. Porous tantalum is an ideal alternative repair material for bone defects because of its excellent biocompatibility and biomechanical properties, which have been previously used in clinical treatment and have achieved good results (Boureau et al., 2015; Hu et al., 2017; You et al., 2019). However, there are few studies on the application of 3D printing combined with porous tantalum materials in repairing bone defects. In this study, 3D printing was used to fabricate a personalized porous tantalum cone to repair bone defects in TKA revision, analyze its early treatment effect, and provide a basis for subsequent wide clinical application.

## Materials and methods

### Participants

Between November 2017 and October 2020, patients who underwent bone defect reconstruction with a 3D-printed porous tantalum cone during TKA revision were followed up. The inclusion criteria were as follows: 1) age >18; 2) patients suffering AORI II bone defects who received TKA revision; 3) detailed preoperative imaging data; and 4) patient and family signed the informed consent form and agreed to participate in this study. The exclusion criteria were as follows: 1) active infection; 2) severe coagulopathy; 3) poor cardiopulmonary function and unable to tolerate surgery; 4) unable to complete postoperative follow-up; 5) allergic to tantalum; and 6) presence of diseases such as malignant tumor that may affect postoperative follow-up. This study was approved by the Institutional Human Ethics Committee (SWH2016ZDCX2010), and all experimental study protocols conformed to ethical norms.

### Study procedures

**FIGURE 1:**
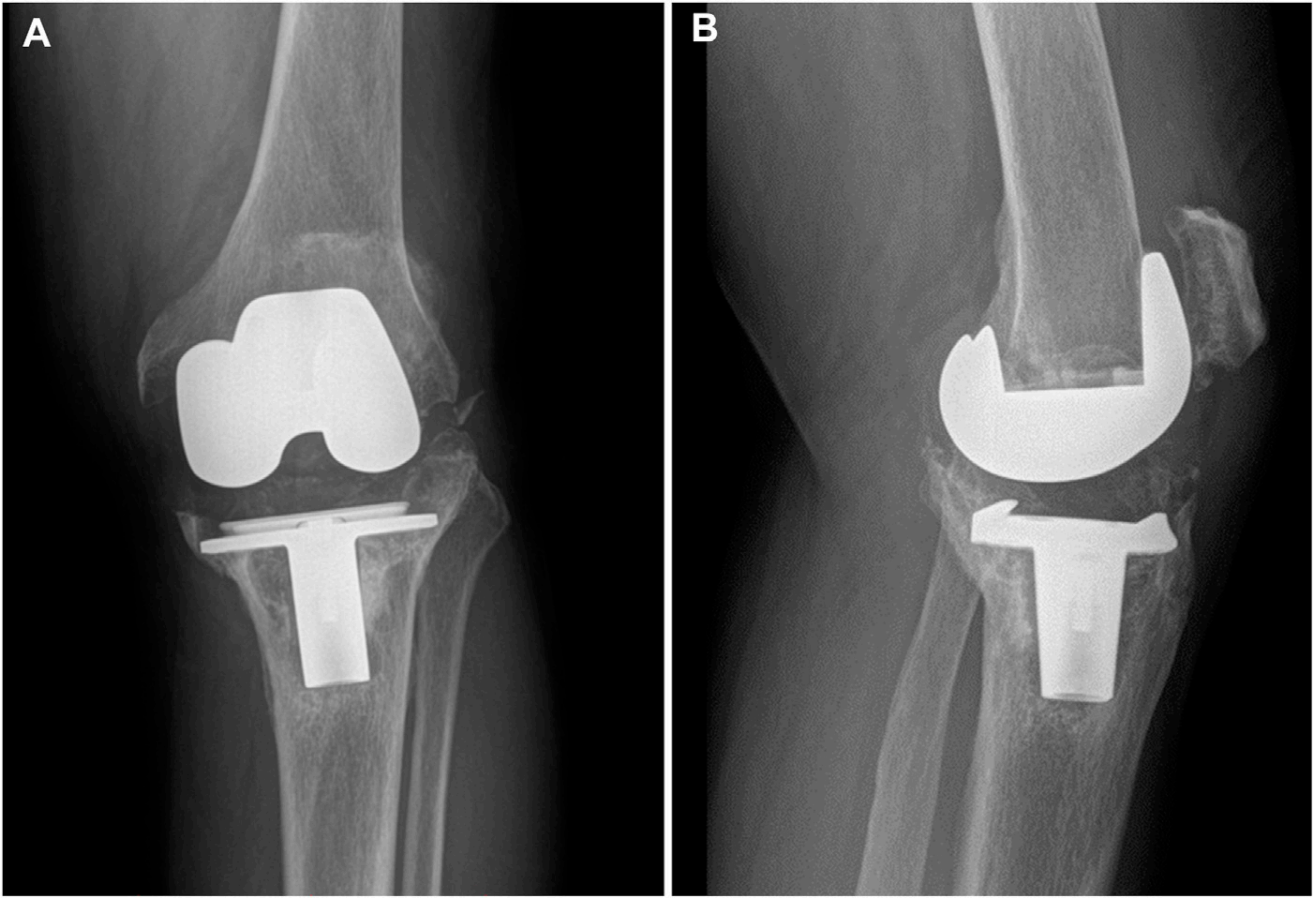
Preoperative radiographs of the typical case showing collapse of the tibial plateau. **(A)** Coronal view; **(B)** sagittal view.

**FIGURE 2:**
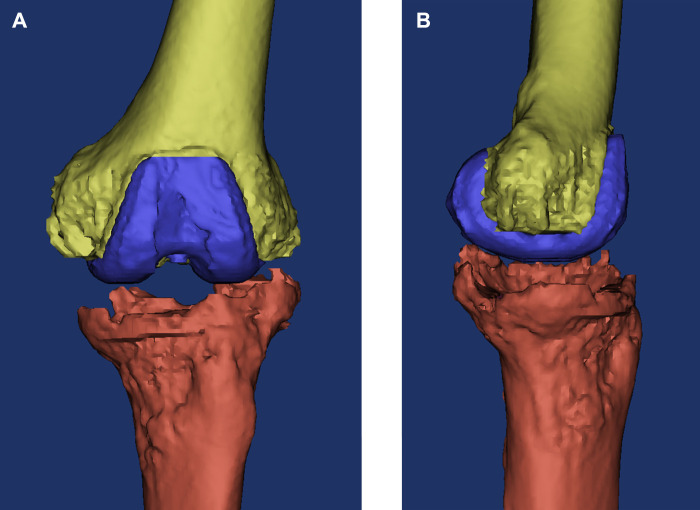
Three-dimensional reconstructed image of the knee based on the CT data before surgery. **(A)** Coronal view; **(B)** sagittal view.

**FIGURE 3:**
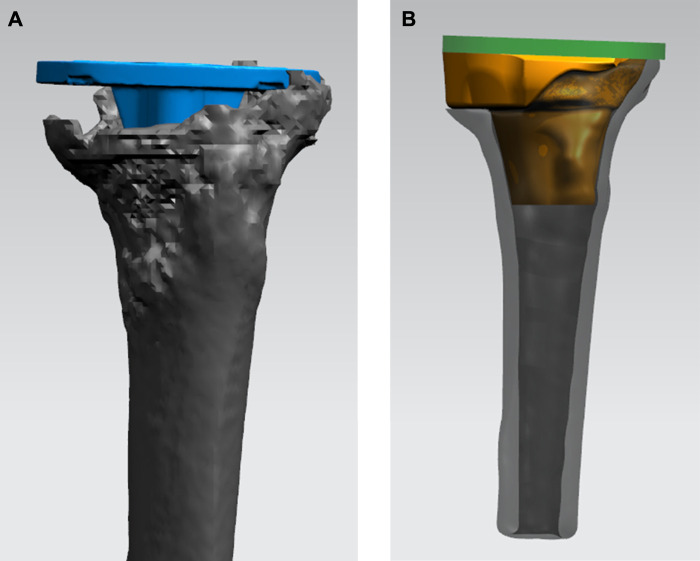
Process of designing the 3D-printed porous tantalum cone. **(A)** Simulating the position of the tibial tray (blue) in the tibia in TKA revision. **(B)** Designing the shape and size of the porous tantalum cone (yellow) conforming to the bone defect.

Main procedures of this study included preoperative imaging examination, prosthesis design and fabrication, surgical operation, and postoperative follow-up. The detailed procedures were as follows: 1) carefully screened the cases that met the inclusion criteria, and obtained the informed consent form; 2) collected the imaging data of the surgical site of the patient, and the professional medical 3D designer performed 3D reconstruction (Figures 1, 2); 3) the designer and the surgeon determined the surgical plan and designed the personalized porous tantalum prosthesis; 4) after determining the prosthesis design, completed the prosthesis printing and sterilization; 5) implanted the personalized porous tantalum prosthesis in TKA revision to repair the bone defect; 6) completed the postoperative follow-up and evaluated the patient’s joint function and various indicators.

### Image data collection

All patients enrolled in this study routinely underwent X-ray and 3D CT scans ranging from the ilium to the ankle, with a scan slice thickness of 1 mm. All digital images were extracted and saved in digital imaging and communications in the medicine (DICOM) format and were uploaded into the Materialise’s Interactive Medical Image Control System (MIMICS 17.0 Software, Materialise Corporation, Belgium) for 3D reconstruction and subsequent prosthesis design.

### Preoperative planning and prosthesis design

The 3D models of the bone defect and surrounding tissues were established in MIMICS software based on the acquired CT scan DICOM data as described previously, and the process of designing the prosthesis was performed by an experienced engineer (Figure 3). In order to make the prosthesis meet actual clinical demands, engineers and surgeons conducted in-depth communication. The main considerations included the following aspects: 1) most of the patients undergoing knee revision were the elderly with osteoporosis, and the bone debris might be removed during operation; 2) the anatomical shape of the prosthesis should be closely matched with the actual bone defect, so that it would have excellent stability and achieve the therapeutic effect of long-term use; 3) the prosthesis had a feature of porous structure, and its weight and elastic modulus should be considered to avoid stress shielding and other conditions; 4) according to the previous study, the optimal porosity should be designed to promote the subsequent bone ingrowth (Guo et al., 2019).

In the process of designing the porous tantalum cone, the bone defect was simulated according to the preoperative CT images of the patient. The design of the prosthesis mainly included three concepts, namely, anatomical matching,mechanical balance and restoration of function. Based on bone defects, different porous tantalum prostheses were designed. With a few modifications, the porosity was designed to be 75%–80%, so that the autologous bone tissue can be closely combined with the implanted cone as far as possible. After the preliminary design of the prosthesis, a finite element model was established for mechanical analysis to ensure that the implanted cone can better disperse the stress without affecting the joint movement.

**FIGURE 4:**
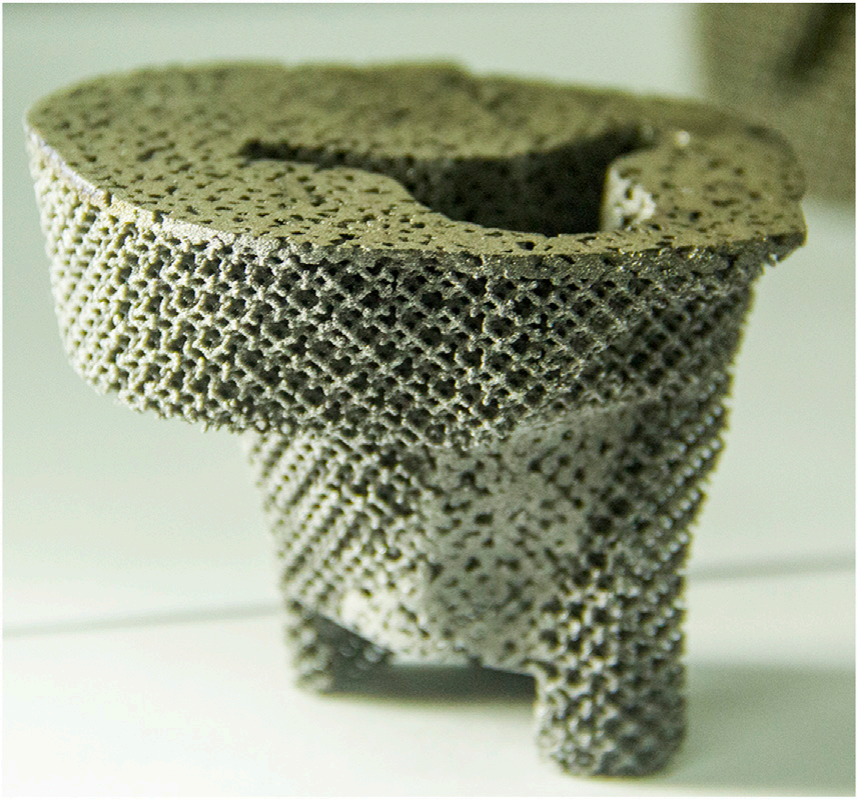
Personalized porous tantalum prosthesis printed by pure tantalum.

After the prosthesis design was completed, the data of the designed prosthesis were converted into the STL format and imported into a 3D printer to print a 1:1 plastic model of the prosthesis and surrounding tissues, and the clinician performed a detailed preoperative protocol simulation. Subsequently, porous tantalum prostheses were fabricated and completely sealed and were stored for operation after disinfection (Figure 4).

### Surgical procedures

**FIGURE 5:**
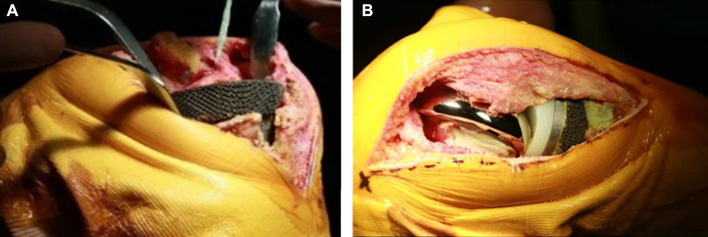
Operation of implanting 3D-printed tantalum prosthesis and the TKA revision surgery. **(A)** After trimming the tibial bone defect, the tantalum prosthesis was implanted into the tibia. **(B)** Subsequently, commercial components used in TKA revision were implanted into the knee.

The patient lied in the supine position. After successful anesthesia, a tourniquet was placed on the root of the thigh at a pressure of 280 mmHg. An anteromedial incision was made in the knee, and the skin, subcutaneous tissue, and deep fascia were incised layer by layer to expose the joint cavity. Due to the bone defect, a great number of wear debris were observed in the knee joint, and no secretion was seen in the joint cavity. The hypertrophic synovium and scar tissue in the joint cavity were removed, and the bone debris inside the joint was debrided. In the position of extreme knee flexion, the femoral and tibial prosthesis was taken out, and cement was carefully removed from the bone interface using a curette. After the removal of cement, the medical pulse irrigator was used to flush the surgical field to reduce the risk of infection. Subsequently, medullary reaming of the femur and tibia was conducted, and the excess cortical bone was cut according to the preoperative plan. A customized 3D-printed porous tantalum cone was implanted into the tibia to reconstruct the bone defect; commercial components (Zimmer, tibial component, LCCK femoral component, and LCCK liner) were also used in the revision (Figure 5). After implanting all prostheses, knee flexion and extension were conducted to confirm reliable prosthesis placement. Anticoagulation therapy and intravenous injection of antibiotics were given 6 h after the revision. A silicone drainage tube was maintained until 24 h postoperatively. Functional recovery exercises began after patient’s awakening from anesthesia, and ankle flexion and extension were performed to prevent lower limb thrombosis; daily knee flexion and extension were performed on the second postoperative day, and the angle of motion was gradually expanded to 90 degrees.

### Indicators

Time of revision, intraoperative blood loss, and postoperative drainage volume were recorded to evaluate the surgical trauma. All patients were followed up at least three times (1, 3, and 6 months after the operation). Preoperative and postoperative visual analog scale (VAS) and Hospital for Special Surgery (HSS) scores were recorded for analysis of improvement in joint function and pain. The 36-Item Short Form Health Survey (SF-36) is an easy measure of reflection in life quality. SF-36 of pre-operation and last follow-up were also recorded to assess the improvement of life quality after revision. At the last follow-up, a CT scan was performed to confirm whether the bone defect was completely reconstructed.

### Statistical analysis

All statistical analyses were performed by SPSS software (version 22.0; IBM Corp, United States). The continuous variables were expressed as the mean ± standard deviation. VAS, HSS, and SF-36 scores were compared before and after the operation using the paired *t*-test. The significance level was set at *p* < 0.05.

## Results

All patients completed postoperative follow-up, and relevant clinical characteristics such as gender, age, diagnosis classification, and disease course are shown in Table 1. All operations were successfully and smoothly completed, and there were no postoperative complications such as infection, implant loosening, and joint dysfunction. The mean operation time was 189.8 ± 34.1 (range, 139–246) min; intraoperative blood loss was 250.1 ± 76.4 (range, 200–400) ml; mean postoperative drainage volume was 506.7 ± 300.8 (range, 100–1010) ml. The mean preoperative VAS score was 7.2 ± 1.1, the mean VAS score was 3.1 ± 0.9 at the last follow-up (*t* = 6.730, *p* < 0.001), and the VAS score was significantly improved compared with the preoperative score. The mean preoperative HSS score was 31.3 ± 5.7, the mean HSS score was 64.7 ± 7.2 at the last follow-up (*t* = 8.111, *p* < 0.001), and there was a significant improvement in joint function compared with the preoperative score. The mean preoperative SF-36 score was 38.8 ± 7.8 and the mean SF-36 score was 77.8 ± 4.2 at the last follow-up (*t* = 9.836, *p* < 0.001). Detailed data are shown in Table 2.

**TABLE 1:** Demographics of patients.

Patient	Age	Sex	AORI	Symptom	Side of operation	Indication for revision	Duration (year)
1	85	M	Type IIB	Pain and dysfunction	Left	Aseptic loosening	17
2	83	F	Type IIA	Pain and dysfunction	Left	Aseptic loosening	11
3	78	F	Type IIA	Pain and dysfunction	Right	Prosthetic joint infection	3
4	68	F	Type IIB	Pain	Right	Prosthetic joint infection	1.5
5	75	F	Type IIB	Pain and dysfunction	Right	Aseptic loosening	10
6	58	M	Type IIA	Pain	Right	Instability	8

**TABLE 2:** Relevant data of operation and follow-up.

Variable	Value
Operation time (min)	189.8 ± 34.1 (range, 139–246)
Intraoperative blood loss (ml)	250.1 ± 76.4 (range, 200–400)
Postoperative drainage volume (ml)	506.7 ± 300.8 (range, 100–1010)
Follow-up duration (month)	26.3 ± 12.6 (range, 9–44)
VAS score (pre. vs. post.)	7.2 ± 1.1 vs. 3.1 ± 0.9
HSS score (pre. vs. post.)	31.3 ± 5.7 vs. 64.7 ± 7.2
SF-36 score (pre. vs. post.)	38.8 ± 7.8 vs. 77.8 ± 4.2

## Discussion

**FIGURE 6:**
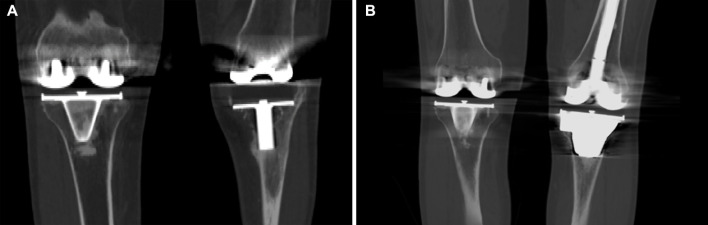
CT images of the representative case showed that the tibial bone defect was effectively reconstructed by the porous tantalum cone. **(A)** Preoperative image; **(B)** postoperative image at 3 years after the operation.

**FIGURE 7:**
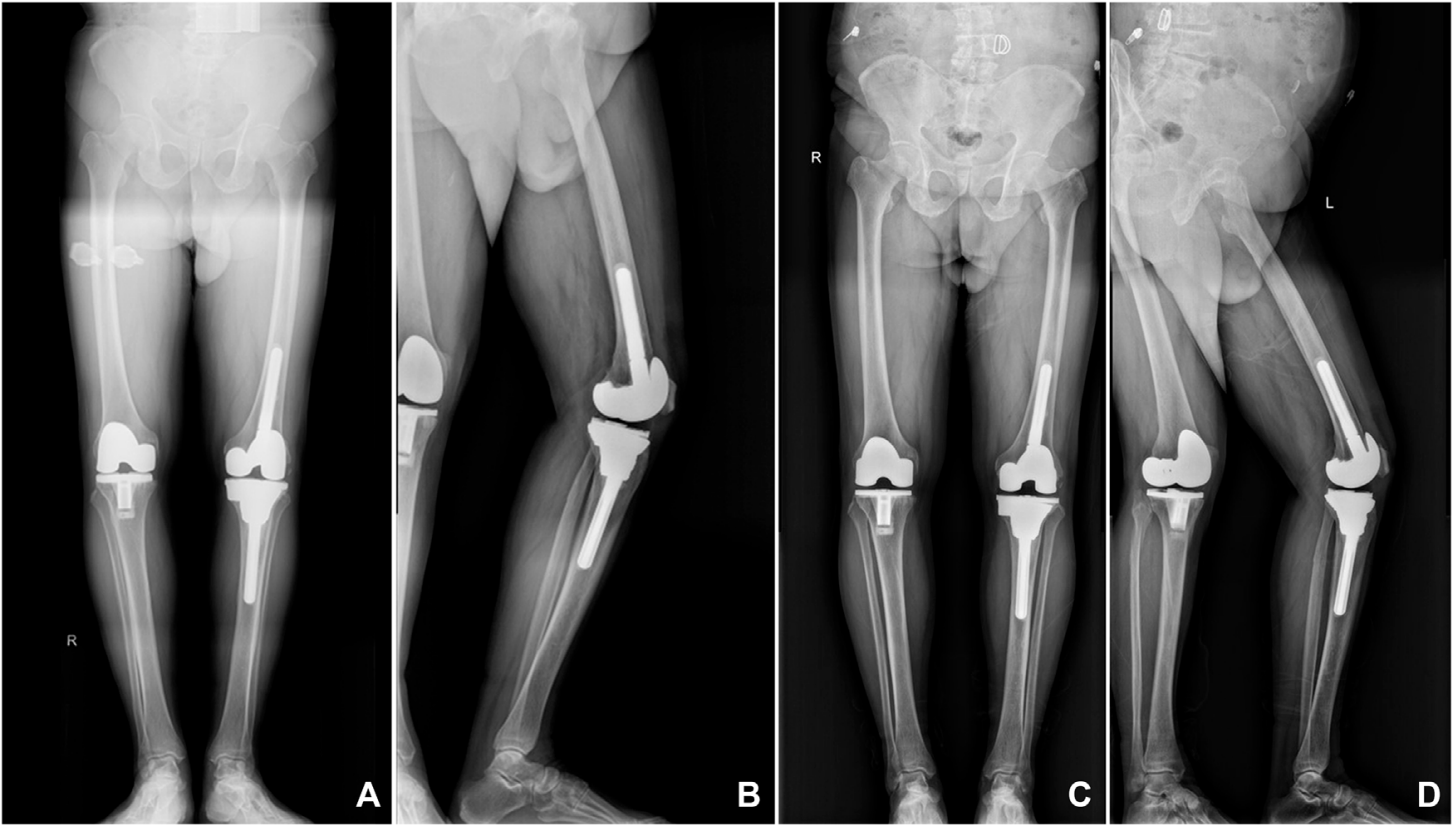
X-ray images of a representative patient showed that the 3D-printed porous tantalum cone is stable and tightly integrated with the surrounding bone tissue. **(A,B)** Postoperative radiographs were taken at 6 months after surgery; **(C,D)** postoperative radiographs were taken at 3 years after surgery.

In this study, we used 3D printing combined with porous tantalum to manufacture a personalized cone for the clinical treatment of metaphyseal bone defects. After multiple postoperative follow-ups, it was confirmed that this technology has a significant clinical therapeutic effect, which can effectively repair the metaphyseal bone defects of the knee joint and improve the joint function of patients (as shown in Figures 6, 7). In TKA revision, most patients are prone to metaphyseal bone defects due to various causes, such as joint prosthesis instability and abnormal lower extremity alignment, affecting normal joint activity and the quality of life (Lotke et al., 2006; Engh and Ammeen, 2007).

In the past, the treatment of metaphyseal bone defect in knee revision was mainly determined according to the patient’s underlying disease, the severity of the bone defect, the reason for revision, and the postoperative knee function and activity expectations. AORI type I bone defects, because of their small depth and area, are mainly filled with bone cement; however, the implanted bone cement may decrease in size, resulting in prosthesis loosening during recovery (Toms et al., 2009). For AORI type II bone defects, allogeneic bone transplantation is another treatment strategy. Clatworthy et al. (2001) used this technique to treat 52 patients with tibial plateau bone defect requiring knee revision. After a long-term follow-up, 75% of patients had effective improvement in joint function. However, a large defect area cannot be effectively repaired; there is also a risk of transmitting diseases and bone graft resorption, and its high treatment cost also hinders its wide application in clinical practice (Dennis and Little, 2005; Rudert et al., 2015).

The metal cone is suitable for bone defects with a large area and has a certain therapeutic effect on metaphyseal bone defects. At present, this method is widely used for the treatment of bone defects in clinical practice. This technique can solve the problem of bone absorption or transmission of diseases (Issack, 2013; Divano et al., 2018). Porous tantalum material has been widely used in many fields of orthopedics because of its excellent biocompatibility, corrosion resistance, and mechanical properties, and the porous structure is conducive to inducing host bone ingrowth and bone adhesion and is the main material of customized metal cone produced by Zimmer (Bencharit et al., 2014; Potter et al., 2016; Huang et al., 2021). Howard et al. (2011) treated patients with bone defects after TKA revision. They used porous tantalum cones to fill femoral metaphyseal bone defects, and after an average follow-up of 33 months, most patients had significant improvement in knee function. Meneghini et al. (2008) also used porous tantalum cones to treat tibial AORI type II and III bone defects in TKA revision and achieved good therapeutic results, but some patients experienced secondary revision. Brown et al. (2015) reported the cases of using porous tantalum cones to fill bone defects and performed joint replacement. After an average follow-up of 40 months, it was found that the treatment effect was good in most patients, with some patients undergoing second operation due to infection and periprosthetic fracture, etc. Lachiewicz et al. (2012) retrospectively analyzed patients treated with porous tantalum cones, and during follow-up, the radiographic findings indicated that the implant augments showed good osseointegration with the host bone. Although the therapeutic effect of the metal cone is better than that of allogeneic bone transplantation, the metal cone is a customized commodity. When the patient’s bone defect is large and irregular in shape, it is difficult to completely match. Large and complex bone defects are the main problems surgeons are confronted with.

In recent years, with the development of precision medicine, 3D printing has also become increasingly progressive. With its advantages of precision and individualization, it is, especially suitable for the design and fabrication of personalized prostheses in orthopedics (Duan et al., 2019; Sun M. et al., 2020). The rise of 3D printing has provided a new and accurate solution for the treatment of various osteoarticular diseases (Duan et al., 2018). England et al. (2021) used 3D printing to manufacture a porous titanium prosthesis for the repair of bone defects during TKA revision. The follow-up results showed that the porous titanium prosthesis had better integration and stability with the host bone and had a better therapeutic effect on bone defects. Sun M. L. et al. (2020) used 3D printing to manufacture personalized surgical guides to assist in TKA, which could make the surgical operation more accurate. Compared with titanium, tantalum has good physical and chemical properties and is more suitable for the repair of bone defects. Balla et al. (2010) used 3D printing to prepare porous tantalum and porous titanium and found that porous tantalum has better biocompatibility than porous tantalum. When osteoblasts were cultured on the surface of porous tantalum, the expression level of alkaline phosphatase was increased, indicating that porous tantalum has a better bone-promoting ability. Guo et al. (2019) found through experimental studies that porous tantalum could promote stem cell proliferation, adhesion, and differentiation more than porous titanium and had better osteointegration performance, which was more suitable for utility as a bone substitute product. For metaphyseal bone defects after TKA, 3D-printed personalized prostheses can better match the shape of the defect and help make a detailed preoperative plan and enable a smoother operation. The porous structure can make the implanted metal more tightly integrated with the host bone, ensure the stability of the implanted augment and the stability of the joint prosthesis, and avoid the occurrence of re-revision (Small et al., 2022).

To the best of our knowledge, it is rare to use 3D printing to manufacture personalized porous tantalum prostheses for repairing metaphyseal bone defects in TKA revision. In this study, 3D printing was used to fabricate a personalized porous tantalum cone for repairing the metaphyseal bone defect, achieving an ideal repair effect, effectively relieving patient’s pain symptoms and improving joint range of motion and the patient’s quality of life. This study also has some limitations: first, due to the small number of patients included in the study, it lacks a control group; second, this study mainly observes the mid-term clinical efficacy, the follow-up time is relatively short, and it still needs a longer follow-up to observe its long-term therapeutic effects; third, a simple radio imaging follow-up was performed after the operation to observe the stability of the prosthesis, but the relevant osseointegration is not analyzed in detail.

## Conclusion

This study reported the mid-term clinical outcome of the 3D-printed porous tantalum prosthesis for reconstructing bone defects during TKA revision. In this study, a porous tantalum prosthesis manufactured by 3D printing presented favorable effects on the treatment of bone defects in revision, including relieving pain and improvement of knee function and quality of life. Great reconstruction of bone defect was achieved by anatomically conforming the design and excellent osseointegration of tantalum prosthesis. Despite these beneficial outcomes, future multicenter case–control studies are still needed to be conducted to research the long-term effect.

## Data Availability

The original contributions presented in the study are included in the article/Supplementary Material; further inquiries can be directed to the corresponding authors.
